# Perceptions of Food among College Students in the Field of Food Science: A Food Sustainability Approach

**DOI:** 10.3390/foods12050917

**Published:** 2023-02-21

**Authors:** Maria Clara de Moraes Prata Gaspar, Claudia Soar, Mari Aguilera, Maria Clara Gomez, Ricard Celorio-Sardà, Oriol Comas-Basté, Cristina Larrea-Killinger, M. Carmen Vidal-Carou

**Affiliations:** 1 Departament d’Antropologia Social, Facultat de Geografia i Història, Universitat de Barcelona (UB), Carrer de Montalegre 6, 08001 Barcelona, Spain; 2 Institut de Recerca en Nutrició i Seguretat Alimentària (INSA-UB), Universitat de Barcelona, Av. Prat de la Riba 171, 08921 Santa Coloma de Gramenet, Spain; 3 Nutrition Post-Graduate Program, Department of Nutrition, Federal University of Santa Catarina, 88040-900 Florianopolis, Brazil; 4 Departament de Cognició, Desenvolupament i Psicologia de l’Educació, Secció Cognició, Facultat de Psicologia, Universitat de Barcelona (UB), Passeig de la Vall d’Hebron 171, 08035 Barcelona, Spain; 5 Institut de Neurociències (UBNeuro), Universitat de Barcelona (UB), Passeig de la Vall d’Hebron 171, 08035 Barcelona, Spain; 6 NeuroDevelop eHealth Lab, eHealth Center, Universitat Oberta de Catalunya (UOC), Rambla de Poble Nou 156, 08018 Barcelona, Spain; 7 Departament de Nutrició, Ciències de l’Alimentació i Gastronomia, Facultat de Farmàcia i Ciències de l’Alimentació, Campus de l’Alimentació de Torribera, Universitat de Barcelona (UB), Av. Prat de la Riba 171, 08921 Santa Coloma de Gramenet, Spain; 8 Xarxa d’Innovació Alimentària (XIA), C/Baldiri Reixac 4, 08028 Barcelona, Spain; 9 Anthropology of Crises and Contemporary Transformations (CRITS), Departament d’Antropologia Social, Facultat de Geografia i Història, Universitat de Barcelona (UB), Carrer de Montalegre 6, 08001 Barcelona, Spain

**Keywords:** sustainable diet, food, perception, eating practices, dietetic, food technology, college students

## Abstract

The complex concept of food sustainability has become crucial in all spheres of life. Dietitians, food scientists, and technologists are in a unique position to promote sustainability in food systems. However, the perceptions of food sustainability among food science professionals and college students are under-researched, particularly in Spain. The aim of this study was therefore to analyze perceptions related to food and to food sustainability in a sample of Human Nutrition and Dietetics (HND) and Food Science and Technology (FST) students in Barcelona (Spain). An exploratory and descriptive cross-sectional study was carried out using qualitative and quantitative methodology and convenience sampling. Two focus groups and an online questionnaire were conducted (300 participants completed the survey, 151 from HND and 149 from FST). Although the students expressed concern about food sustainability, their dietary choices were primarily associated with or influenced by taste/pleasure and health/nutrition. The issue of sustainability seemed more internalized by women than men, whereas the generalized conception of a sustainable diet was essentially based on environmental aspects, with socioeconomic dimensions largely overlooked. The concept of sustainability should be promoted among food science students in all its multidimensionality, and actions need to be implemented that bring sustainability closer to students’ social practices, which should be incorporated into all university education and that is taught by professors duly trained in the subject.

## 1. Introduction

Food sustainability is a broad, multidimensional, and complex concept that is difficult to define [1]. It attempts to reconcile public health and ecological discourses but also involves the economic, social, and cultural dimensions of food [2]. According to the Food and Agriculture Organization (FAO), “*sustainable diets are those diets with low environmental impacts which contribute to food and nutrition security and to healthy life for present and future generations. Sustainable diets are protective and respectful of biodiversity and ecosystems, culturally acceptable, accessible, economically fair and affordable; nutritionally adequate, safe and healthy; while optimizing natural and human resources*” [3].

Sustainability has become a key concept in all spheres of social, cultural, economic, and political life [4]. On a global level, the definition of the Sustainable Development Goals in 2015 by the United Nations, followed by the presentation of the European Green Deal in 2019, made sustainability the central axis of strategies aimed at improving human living conditions and the environment. In this context, food systems have been increasingly associated with environmental, economic, and social impacts that directly affect the sustainability of the planet [5]. Public and private agents have developed actions and multiplied recommendations to integrate sustainable consumption into daily life [6,7]. Due to its direct links with human and environmental health, sustainability has also become inseparable from discourses on healthy eating. In the 1980s, Gussow and Clancy (1986) [8] highlighted the importance of the connection between nutrition, health, and the environment. More recently, this link was also evidenced in a report issued by the EAT-Lancet Commission [9].

Social perceptions, which are based on subjective and socially elaborated knowledge, influence social practices. Although there are official definitions and institutional recommendations regarding sustainability [10,11], individuals perceive this concept in multiple ways [12,13]. Food professionals, such as dietitians, food scientists, and technologists, are in a unique position to influence sustainability at different stages of the food chain (i.e., from production to consumption) by developing production techniques and/or promoting sustainable food practices [14,15,16,17]. Although in recent years more and more studies have explored perceptions of food sustainability among lay individuals [6,12,18,19,20], little research has addressed this issue in professionals from the field of food and nutrition [21].

In a study of US dietitians, Hawkins et al. (2015) [16] reported that over 45% of survey participants agreed that climate change is an important issue and should be considered in practice-related behaviors. However, it was found that only 8% of dietitian workplaces provided funding for diet-related climate change mitigation activities. A survey of American nutrition and dietetics programs showed that 68% of educators were interested in sustainability education techniques, but felt inadequately prepared to put them in practice [22]. 

Undergraduate students are at a crucial stage of professional training. As a young population, they are potentially open to absorbing new trends and constitute a highly suitable collective to receive training aimed at fostering sustainability [6], especially students enrolled in the field of food science [21]. Burkhart et al. (2020) [21] observed that Australian dietetic students were familiar with and concerned about sustainability, but only in a superficial way.

To the best of our knowledge, despite the importance of sustainability for professionals working in food and nutrition, very few studies have hitherto analyzed the social perceptions of sustainability-related issues among professionals or students in this field. Most of the available research has assessed the level of familiarity with the concept of sustainability or the importance attached to it among dietetic professionals or college students, mainly in an Anglo-Saxon context. In Spain, the perceptions of sustainability among these groups have been little explored. Understanding social perceptions among food science professionals or college students is fundamental to improve their academic training, promote a more critical perspective of the food system, and ultimately promote sustainability [21,22,23]. In this context, the aim of this study was to analyze the perceptions of food and food sustainability among college students of Human Nutrition and Dietetics (HND) and Food Science and Technology (FST) in Barcelona (Spain).

## 2. Materials and Methods

### 2.1. Setting and Sample

An exploratory and descriptive cross-sectional study aiming to analyze perceptions of food among college students using both qualitative and quantitative methodology was carried out between May 2020 and September 2021 by an interdisciplinary team.

The study was conducted with a convenience sample of male and female college students enrolled in any of the four years of bachelor’s degrees in HND and FST at the University of Barcelona (UB), a reference institution in these fields in Spain. According to the UB, the HND bachelor’s degree trains professionals capable of developing activities aimed at feeding the individual or groups of individuals to attend to their physiological or pathological needs, taking into account the principles of health protection and promotion, disease prevention, and dietary and nutritional treatment. On the other hand, the training in FST is essential for the growth, improvement, and diversification of the food industry, which must respond to new needs, concerns, and conveniences of today’s society in the food field. No exclusion criteria were established with respect to the age of the participants, place of residence, or nationality. To characterize the sample, data were collected about educational level, year of education, gender, age, municipality of residence, type of cohabitation, work activity or internship, parental level of education, and average monthly household income. 

### 2.2. Data Production and Analysis

A fully structured questionnaire was developed specifically for the study (see Supplementary Material) based on data obtained in the qualitative research phase, as well as from previous studies on the food perceptions of HND college students and/or dietitians [24,25,26] and the general population [27,28,29].

The qualitative phase was based on focus groups, a useful technique in the exploratory stages of investigation that collects data through group discussions on a specific topic proposed by the researcher [30]. The resulting insights serve as a resource for understanding social representations and deepening research questions [31]. Two focus groups were conducted with a total of 13 students (11 females): five from HND and eight from FST. Participants were recruited by sending an email announcing the project to all students enrolled in these courses, and all those who responded positively participated in the focus groups. Due to restrictions related to the COVID-19 pandemic, the two focus groups were held online in October/November 2020 via the Zoom platform. The participants were equipped with a microphone and a video camera. 

The 90-minute focus groups were led by an anthropologist specializing in food studies and qualitative techniques, and observed by a student with a master’s degree in anthropology and ethnography. The focus group discussions were guided by an interview script specifically drafted for this study, which was composed of open questions about perceptions related to food. The resulting data were later incorporated into the instrument used in the quantitative phase to improve accuracy and constituted a complementary part of the overall results. The focus group discussions were recorded with the consent of the participants and were transcribed verbatim. 

The discussions were analyzed on a thematic basis [32] by coding the content in analytical categories defined according to the objectives of the study and the emerging discourses: perceptions of healthy eating/food, meanings of food, trust/distrust of food, food sustainability, culinary activity, food choices, vegetarianism, and changes in perception while studying the degrees. Two researchers independently read the transcripts to identify the categories, the results were compared, and the final categories were determined after discussion between the researchers. Finally, the coding and systematization of the data was carried out using the qualitative data analysis software Atlas-Ti (version 8. *Visual Qualitative Data Analysis*. 2017). 

Based on the insights obtained from the qualitative stage, a questionnaire was developed. The questionnaire was sent for review to 20 experts from the fields of nutrition (12), statistics (1), anthropology (5), and sociology of food (2), and adjustments were made based on their suggestions regarding question clarity, relevance, and pertinence. A pilot test was carried out with 30 students who were invited to comment on the questionnaire after completing it. The final questionnaire contained 31 multiple choice or Likert scale questions (including those for the sample characterization) addressing the perceptions related to food, healthy eating, and sustainable issues. In addition, it contained an open-ended question: “Which word do you associate with the concept of “food”? (Indicate one word only).” For the analysis of perceptions regarding food sustainability, five questions were selected from the questionnaire: besides a free-association task and self-assessment of diet, they were focused on aspects influencing food choices, food concerns, and what contributes to a sustainable diet. 

The questionnaire was administered online between April and May 2021 via the Survey Monkey website. A total of 385 responses were obtained, 85 of which were excluded because they were incomplete, leaving 300 complete responses (78.0%).

For the analysis, second- and third-year students were grouped together, resulting in three groups (1st-, 2nd/3rd- and 4th-year students). All data derived from the questionnaire were entered into SPSS v. 24 for statistical analysis. Descriptive results were expressed as means and standard deviations or frequencies according to the nature of the data. Textual data collected with the free-association task were pre-processed to reduce data dispersion and synonyms, multi-words were identified, and verbs were reduced to infinitives. The final corpus, which included 42 words, was codified into analytical categories defined by the authors. Statistical results were obtained by comparing the distribution of response frequencies using the chi-square statistic, which was considered significant when <0.05.

## 3. Results

### 3.1. Participant Profile

Table 1 summarizes the general characteristics of the college students who completed the questionnaire. Out of the 300 participants, 151 were studying HND and 149 FST, representing 47.0% and 45.0% of the total enrollments in the two bachelor’s degrees, respectively. All the participants were students of the University of Barcelona, and all lived in Catalonia (Spain). The overall mean age was 21.25 (±3.16) years (21.76 ± 3.80 for the HND students and 20.73 ± 2.24 for the FST students). Most of the informants were female (80.3%) (χ2 = 11.53, *p* = 0.030). The monthly household income of about 50% of the sample was 2000–4999 euros. The parents of 57.4% of the participants had received at least higher education. In total, 59.3% of the participants were not in employment, and the proportions differed between the two degrees (χ2 = 8.17, *p* = 0.010). For example, a higher percentage of the HND students reported doing an internship.

### 3.2. Food Perceptions and Choice

**Figure 1:**
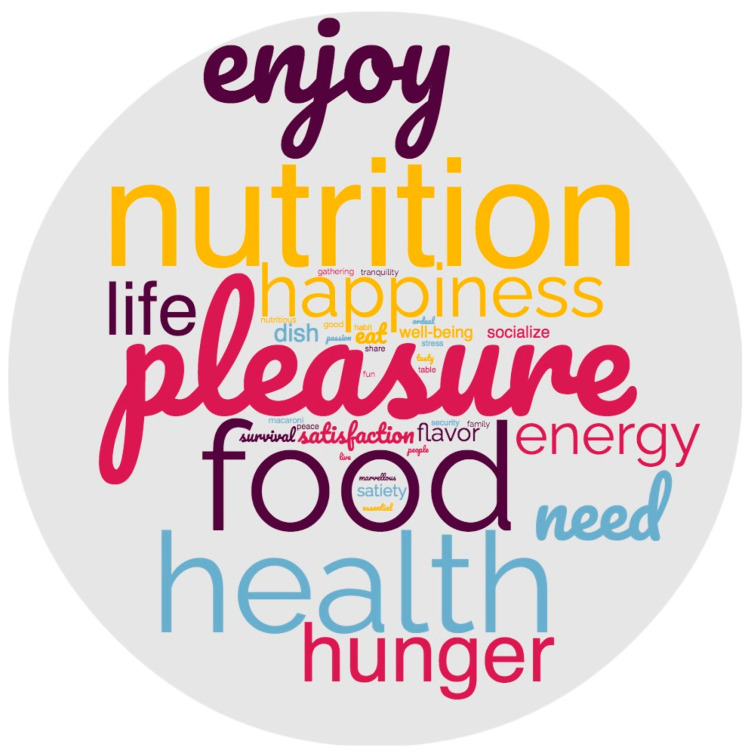
Word cloud based on associations with food. Larger font means higher frequency of mentions. Different colors here are used for better clarity of the different words, they don’t follow a specific pattern.

In the free-association task, no words from the semantic field of sustainability were associated with “food”. The most-cited words were related to pleasure, nutrition, and health (Figure 1). The qualitative phase of the study confirmed this dual perception of eating—an act with a physiological/nutritional dimension that also confers pleasure:

*“The first thing is to get nourishment, nutrition, but then also to enjoy the food as such, uh... to share it, as a means of social connection”*

(Carlos, HND).

The categorization of words into analytical categories revealed that while the category “food” was the most frequent among the FST students (36.9% FST versus 19.3% HND, χ2 = 11.66, *p* = 0.001), the “hedonic and gustatory” dimension was the most recurring among the HND students (41.3% versus 28.9% in FST, χ2 = 4.90, *p* = 0.026) (Table 2). No significant differences were observed in student perceptions according to degree year or gender. 

**Figure 2:**
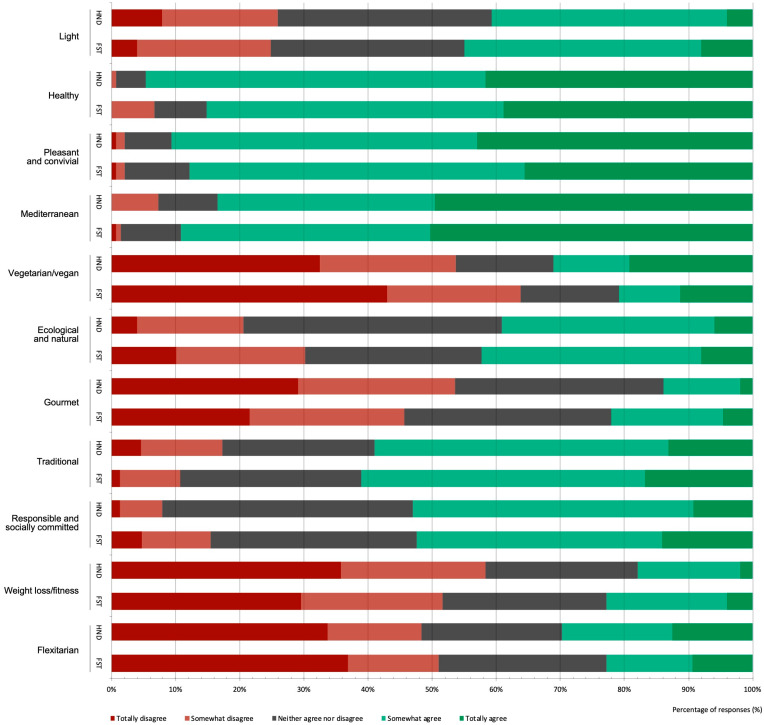
Self-assessment of eating practices: the level of agreement with different diet typologies among college students pursuing bachelor’s degrees in Human Nutrition and Dietetics (HND) and Food Science and Technology (FST), showing the distribution (%) of responses.

The students assessed their eating practices (Figure 2) by indicating levels of agreement (somewhat or totally agree) with different diet typologies. The option “healthy eating” was the most prevalent (90.0%), being statistically higher in the HND students (χ2 = 9.88, *p* = 0.021). This was followed by “pleasant and convivial” (89.3%) and “Mediterranean” (86.3%), the latter being statistically higher among the FST students (χ2 = 9.77, *p* = 0.044). Between 40% and 50% of the students agreed (somewhat or totally) with the dietary patterns related to sustainability: “ecological and natural”, and “responsible and socially committed”. Moreover, 26.0% of the students described their diet as vegetarian/vegan (somewhat or totally), which may be associated with a greater awareness of the sustainability concept, as observed in the focus groups:

*“I don’t eat meat and when I avoid certain foods, I do it because of that, because of sustainability or the origin, things like that. (...) I started when I was very young because of the animals, the little animals. Now, it’s because of sustainability and, to some extent, inertia”*

(Amanda, HND).

*“Sustainability, that’s why I started and why I continue [to avoid eating meat]. In other words, that’s why I’m not very strict, because I understand that maybe [eating] a small amount of meat won’t change the current situation of the meat industry. But it’s true that I limit it a lot and I try to make those around me aware of the carbon footprint of meat consumption”*

(Elena, FST).

No statistical differences were found in the self-perception of dietary practices according to degree year. However, among the advanced HND students, the tendency to describe their diet as “ecological and natural”, “pleasant and convivial”, or “gourmet” was lower compared to first-year students, while agreement with “light” as a descriptor increased. Among the advanced FST students, there was a decline in the choice of “pleasant and convivial”, and “responsible and socially committed”.

**Figure 3:**
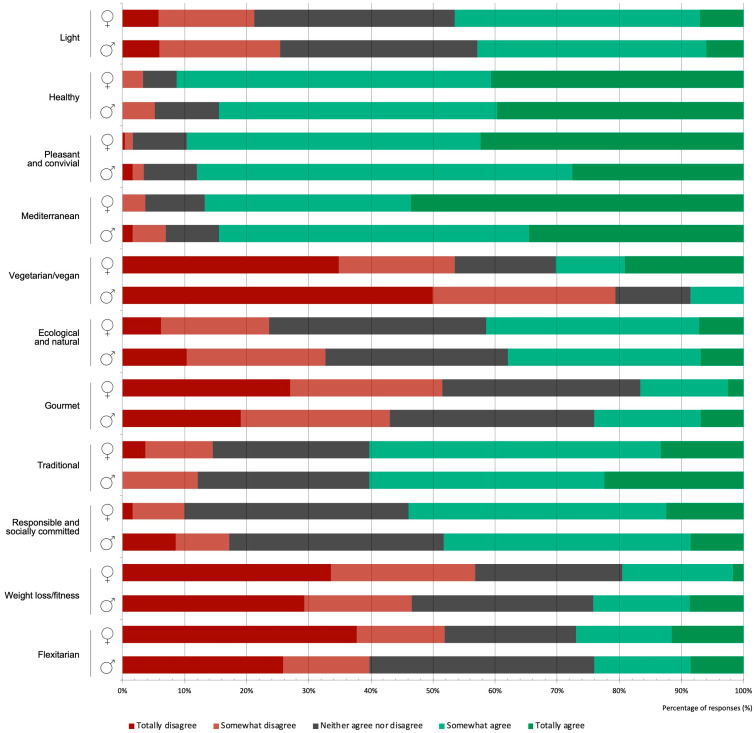
The level of agreement with different dietary typologies in the student dietary self-assessment according to gender, showing the distribution (%) of responses.

Female participants were statistically more likely to describe their diets as “light” (χ2 = 15.25, *p* = 0.004) and “vegetarian/vegan” (χ2 = 17.26, *p* = 0.002) (Figure 3).

Regarding the aspects that most influenced food choices, “pleasure and taste” and “nutritional composition” were the most important (Table 3). Taste and pleasure were also the most frequently highlighted aspects in the focus groups in several cases alongside other elements:

*“I think I’m a mixture of everything because I’m influenced by the taste something has. Obviously, [I’m aware of] the impact it’s going to have on the environment, but I’m also very much a person who takes whatever happens to be in the fridge, especially as I live with my parents. And as my grandparents were farmers, they have a garden and we have a lot of products from my grandparents’ garden, so I eat a lot of what’s in season … so I think the factor that most determines what I eat is whether it’s in season or not”*

(Gemma, HND).

*“I’m trying to cut down on calories a little, I try not to eat too many carbohydrates and I also try to eat as little processed food as possible (…) this seems obvious to me, for health reasons”*

(Samanta, FST).

“Ecology, environment and animal welfare” was the third selected aspect, but with much less frequency. However, according to some participants in the focus groups, food and the quality of food may also be chosen with the aim of reducing the environmental impact. Several strategies may be implemented, considering the type of food (especially animal products), the season, the type of production (whether it is organic or a genetically modified organism, the degree of naturalness), and its origin, mainly if it is local:

*“I try not to eat genetically modified food, I make sure it’s organic, or at least that there are no genetically modified organisms or excessive pesticides, herbicides, that it’s not agribusiness”*

(Amanda, HND).

*“I try to have a little bit of sustainable food. I also try to look at where everything comes from”*

(Samanta, FST)

*“I try not to consume [genetically modified organisms], and I go to markets and look for local products”*

(Alba, FST)

Pleasure and taste preferences were the most important aspects when choosing food for the first-year HND students, while nutritional composition became more important in later years. Furthermore, a statistical difference was observed for “state of mind”: 4.3% of the first-year students chose this option, but it was irrelevant in the decisions of the second-, third-, and fourth-year students (χ2 = 9.09, *p* = 0.011). Additionally, the time of availability gained more influence (1.1% in 1st year and 11.6% in 4th year) (χ2 = 9.57, *p* = 0.008). Similar trends were found among the FST students, for whom nutritional composition became more important in later years (χ2 = 9.83, *p* = 0.009).

“Pleasure and taste” and “nutritional composition” were the aspects that most influenced the food choices of both female and male students, whereas “ecology, environment and animal welfare” was statistically more important among women (Table 3). 

### 3.3. Food Concerns

**Figure 4:**
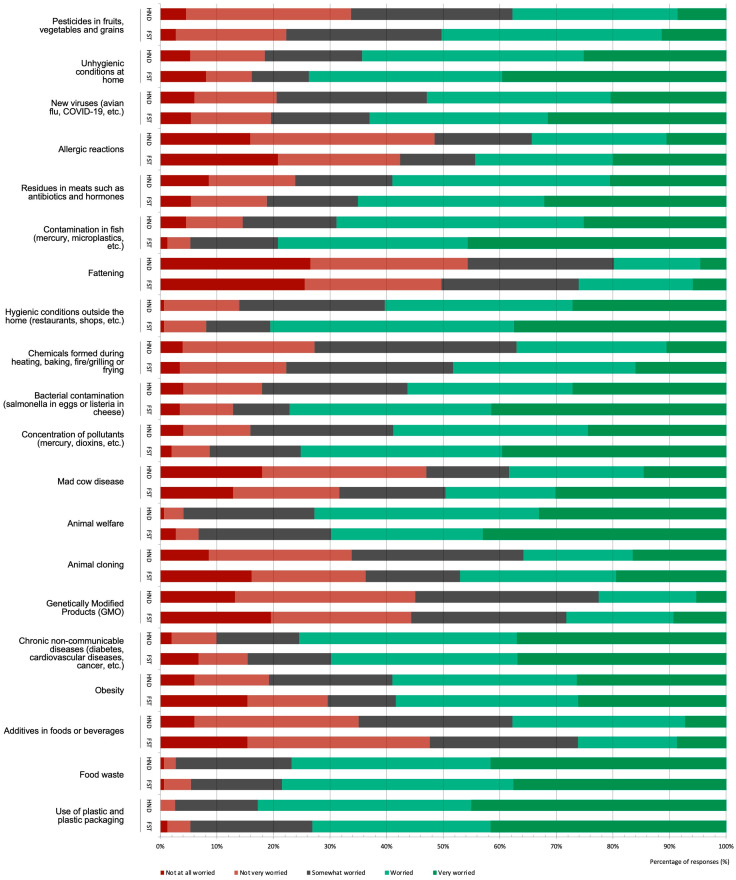
The level of concern for different food-related issues among college students pursuing bachelor’s degrees in Human Nutrition and Dietetics (HND) and Food Science and Technology (FST), showing the distribution (%) of responses.

The food-related aspects that most concerned the students, according to the frequency of “worried” and “very worried” responses, were plastic use (78.0%), food waste (77.7%), and fish contamination (74.0%), the latter being statistically higher among the FST students (χ2 = 17.40, *p* = 0.002). Those of least concern were fattening (23.0%), genetically modified products (25.3%), and additives (colorants, preservatives, and flavorings) (32.0%), the latter being statistically higher among the HND students (χ2 = 12.05, *p* = 0.017) (Figure 4). 

Additionally, compared to the HND students, the FST students showed statistically higher levels of concern for hygienic conditions outside of home (χ2 = 15.28, *p* = 0.004), hygienic conditions at home (χ2 = 10.86, *p* = 0.028), contamination by bacteria (χ2 = 17.26, *p* = 0.002), concentration of pollutants such as mercury and dioxins (χ2 = 14.30, *p* = 0.006), mad cow disease (BSE) (χ2 = 14.30, *p* = 0.006), animal cloning (χ2 = 12.76, *p* = 0.012), and allergic reactions (χ2 = 9.48, *p* = 0.050). Conversely, the HND students showed statistically higher levels of concern for obesity (χ2 = 10.57, *p* = 0.032).

The analysis revealed a decreasing level of concern in the more advanced students of the HND degree for most of the aspects and a transformation in concerns, although only two differences were significant. There was a reduction in concern about the concentration of pollutants: in the first year, 74.5% of the students were worried or very worried, as opposed to 41.9% of the fourth-year students (χ2 = 17.95, *p* = 0.022). Concern about additives used in food or beverages also decreased: 57.5% of the first-year students were worried or very worried, compared to only 18.6% from the fourth year (χ2 = 22.07, *p* = 0.005). Similarities were observed between the HND and FST students, but the trends in the latter were less linear. It is notable that the issue of additives was frequently mentioned by students in the focus groups, especially by the FST students, who described becoming less concerned after beginning their academic training:

*“The issue of additives has changed a lot, because I used to be one of those people who went to supermarkets and saw a product with additives and others without additives, and bought the one without additives, but now I understand that if they are there, it’s for a reason. And not only that they are there for a reason, but that sometimes it is better that they are there than not”*

(Eric, FST).

*“Before I started the degree, I regarded it as something more negative, and [now] I understand that it’s very regulated, that we have a lot of food legislation and we know that in the European Union, EFSA is regulating it”*

(Elena, FST).

**Figure 5:**
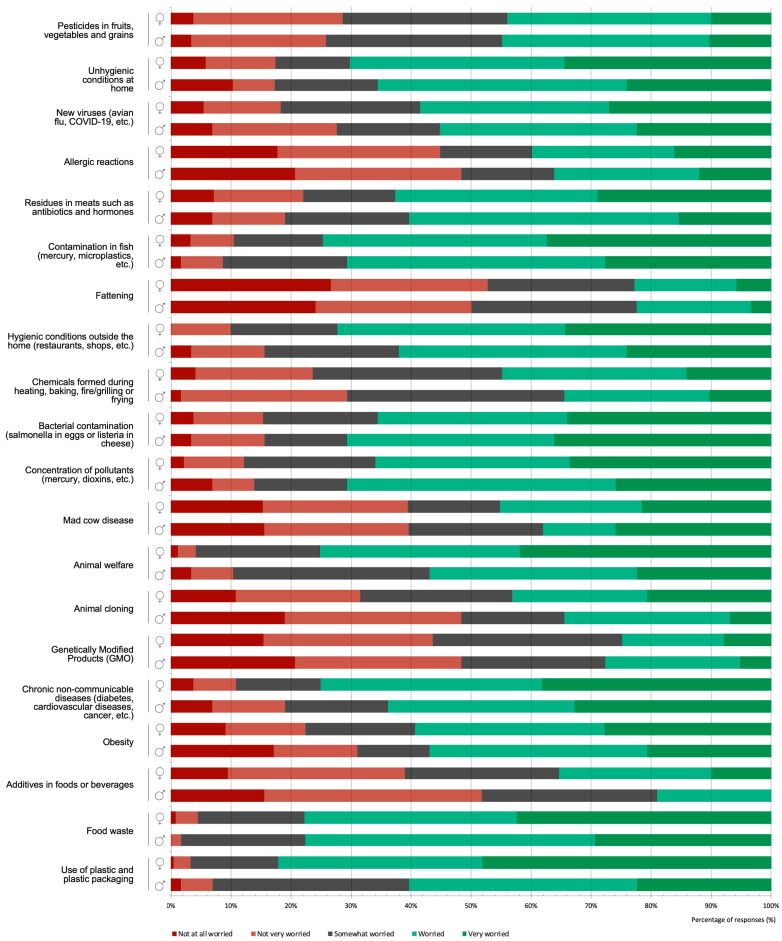
The level of concern for different food-related issues among college students pursuing bachelor’s degrees in Human Nutrition and Dietetics (HND) and Food Science and Technology (FST) according to gender, showing the distribution (%) of responses.

Women showed higher levels of concern for almost all aspects analyzed (Figure 5). Their main cause for concern was the use of plastics and plastic packaging (82.1%, sum of “concerned” and “very concerned”), which was statistically higher than in men (χ2 = 17.89, *p* < 0.001), followed by food waste (77.5%) and animal welfare (75.1%), also statistically higher (χ2 = 10.98, *p* = 0.027), and chronic non-communicable diseases (75.1%). In the case of animal welfare, the results corroborated the data obtained in the focus groups. The main issue of concern for men was food waste (77.6%), followed by contamination by bacteria, the concentration of contaminants such as mercury and dioxins, contamination in fish (all 70.7%), and unhygienic conditions at home (65.5%). Statistical differences, always with higher indices for women, were also found for hygienic conditions outside home (restaurants, shops, etc.) (χ2 = 10.56, *p* = 0.032) and animal cloning (χ2 = 10.83, *p* = 0.028).

### 3.4. Perceptions of a Sustainable Diet

The three most important aspects for a diet to achieve sustainability were judged to be, in order of importance, not wasting food, consuming Km0 or local products, and consuming fresh and seasonal products. Most of the other elements were chosen by less than 5% of the students of either degree (Table 4). Using biodegradable or compostable materials was selected significantly more frequently by the FST students.

In the HND degree, statistically significant differences were found in the conceptions of a sustainable diet among students of different years. New students attributed more importance to consuming organic products (9.2%, χ2 = 27.51, *p* < 0.001), while this aspect almost disappeared among the second-, third-, and fourth-year students (<0.5%). Additionally, there was an increase in the importance attached to “not wasting food” (17.0%, 24.0%, 26.4%, χ2 = 9.01, *p* = 0.011) among the more advanced students. No significant differences were observed between the years in the FST students.

Male and female participants shared similar conceptions of what is necessary to achieve a sustainable diet. However, following a Mediterranean diet was significantly more important for male participants, as was following a vegetarian diet and/or reducing the consumption of animal products. This difference was notable, as female students attached more value to the principles of a vegetarian diet and were more against animal products in all other questions of the questionnaire.

## 4. Discussion

Understanding social perceptions of sustainability is critical for guiding a transition towards more sustainable diets [33]. Although studies on perceptions of food sustainability among young people and university students have been carried out [34,35], to the best of our knowledge, this is the first among college students in the field of food science in Spain. This population is of great importance due to their potentially influential role in food sustainability practices in the future [17]. Based on their skills, curriculum, and involvement in food and nutrition, they may act as promoters of climate change mitigation and other strategies vital for planetary health [9,16]. Food technologists, for example, can promote more sustainable practices along the whole food chain to produce food in a more sustainable way. Dietitians can make food recommendations taking into account the principles of sustainability, such as advising the consumption of fresh, seasonal, and local products, as well as promoting the use of methods to reduce food waste.

Regarding the participant characteristics, the average age was similar to that of other studies with university students [36,37,38]. Focusing on a sample of younger individuals provides insights into the construction of knowledge, and access is gained to environments where certain trends circulate. Notably, this generation of students grew up in the context of increasing environmental, social, and ethical awareness of issues related to food and the climate crisis [6,39]. A statistical difference between genders was expected, as HND students are predominantly women [37,38].

### 4.1. Social Perceptions Related to Food and Sustainability

Understanding perceptions of food and food choices is complex because of the diversity of factors involved, but also crucial, considering the impact of food on sustainability [33,40]. The free-association task revealed that the students mainly associated food with hedonic and nutritional dimensions. When describing their own eating practices and what influences their food choices, the students attached far more importance to pleasure, nutrition, and health than aspects related to sustainability. In the qualitative phase of the study, almost all participants directly or indirectly mentioned taste and/or food preferences as the main aspects conditioning their dietary practices. Compared with the FST students, the HND students were more likely to define food using words related to pleasure and taste, and they also regarded their diet as healthier. Additionally, the food choices of the HND students were more influenced by “disease prevention and health effects”. These differences may be associated with the profile of each bachelor’s degree, as the HND students receive training more “holistic”, less technical, but also more focused on health and healthy eating issues.

The central role of pleasure and taste in the students’ relationship with food is not surprising, as the same perception is found among the general Spanish population [41] and those who cook in Spanish households [42], and fundamentally guides food choices in Spain [43]. These results also corroborate data obtained on a European level, which reveal that Europeans prioritize taste, food safety, and cost over sustainability [44]. However, it is noteworthy that pleasure and taste seemed to become less important for more advanced students, indicating that their food perceptions may have been changed by their training. 

In the contemporary context of the medicalization of food [26,40], the association between food, health, and nutrition is becoming increasingly internalized by the general population in different contexts [26,45], especially among individuals from privileged social classes [46] and women [47,48]. As these groups comprise the majority of the study sample, the observed association of food with health and nutrition could be expected. Available data indicate that the overall Spanish population attaches a similar importance to these aspects rather than those associated with sustainability [49]. In the case of food science students, the relationship between food and nutrition/health may be even stronger given their academic curriculum. A study of Brazilian, Spanish, and French dietitians reported that their training increased concern for health and nutrition [50]. In the present study, the qualitative and quantitative analysis revealed a similar transformation in students as they progressed in their degrees. 

A low percentage of students signaled that the effects on body shape were a dietary choice criterion, and gaining weight was the element that least concerned the participants. In the focus groups, only one female participant revealed that she dieted with the aim of changing her body. This result is perhaps surprising, considering that young people, especially females, are more likely to suffer body image dissatisfaction and go on slimming diets [51,52]. This tendency may be higher among HND students, who are reported to associate their profession with a thin body model [25], and to be at higher risk of eating disorders [53,54,55]. 

The main food-related concerns expressed by the students were issues associated with sustainability, such as the use of plastic and plastic packaging, food waste, and contamination of fish. Therefore, while sustainability was not prominently associated with dietary practices or given as a reason for food choices, it was the subject of a high level of concern when considered a separate category. These results indicate a discrepancy between concerns and practices, the latter being more influenced by aspects such as pleasure and taste. Other studies have also shown that environmental worries are not necessarily translated into more sustainable practices [56,57]. These data may suggest that sustainability, as a dimension of food practices or a significant factor in the relationship with food, is in transition and is not yet a hegemonic discourse among the study participants. 

The concerns expressed by the students were also observed in a Eurobarometer survey [49] in which contamination of fish, meat, or dairy products featured prominently. However, other concerns raised by European and Spanish populations were not important for the student cohort, such as the use of pesticides on fruits and vegetables, the use of additives, or the presence of antibiotics and hormones [49,58]. Although both degrees studied by the participants were in the field of food science, they differed in the type of training and professional activity involved. This was reflected in the higher concern shown by FST students for hygiene and contamination issues, whereas HND students attached more importance to chronic diseases and obesity. 

The results of this study indicate considerable consensus on what constitutes a sustainable diet. Among students of both degrees, regardless of gender, the aspects most frequently chosen were “not wasting food”, “consuming Km0 or local products,” and “consuming fresh and seasonal products”, all of which receive extensive coverage in the Spanish media. Sustainability was essentially associated with environmental issues, with few students considering aspects related to social and economic dimensions, such as “consuming fair trade products” or “buying products directly from the producer”. These results corroborate those of other studies in various countries, including Spain [11,19,59], which report that common perceptions of a sustainable diet do not encompass the complexity and multidimensionality of the concept [1], as defined by the FAO. Compared with the first-year students, the more advanced students did not show a significantly more holistic conceptualization of food sustainability. Certain trends were observed; for example, fourth-year students attached less importance to consuming organic food and were more concerned about food waste but showed little awareness of the relevance of sociocultural aspects of sustainability. In a study in Australia, Burkhart et al. (2020) [21] analyzed the level of familiarity of nutrition students with concepts related to food sustainability and found that they were largely unaware of its association with social development, economic resilience, and cross-cutting issues. The results were considered unsurprising, given the priority assigned by the media to environmental aspects of sustainability and the lack of emphasis placed by nutrition training on social, economic, and political issues. Therefore, the promotion of a more global, critical, and complex view of sustainability in this field would be desirable.

In the present study, the aspects the students most associated with a sustainable diet are in line with the conceptions held by the general population and are the result of contemporary phenomena (e.g., industrialization, urbanization, globalization) that generate certain perceptions and concerns regarding food [40,60]. The element considered most important was “not wasting food”, which increased in significance for more advanced HND students. Food waste is gaining prominence in political, media, and academic discourse as a key issue in the contemporary food system [61,62]. Indeed, reducing food waste constitutes one of the targets of the United Nations Sustainable Development Goals (Goal 12). In Spain, both the Spanish state and the autonomous community of Catalonia have passed laws in the last two years aimed at reducing food waste by 50% throughout the food chain by 2030. Although people in Spain are making efforts to apply measures to reduce food waste [20], three out of four Spanish households in 2020 were still wasting food to some extent [63]. Verdugo et al. (2020) [64] analyzed food waste among Spanish university students and found that it corresponds to 14.5% of the food on a plate. In the present study, the food science students at least showed awareness of the problem. Therefore, even though there may be discrepancies between the norms/perceptions held by this population and their actual practices [40], the results could be regarded as promising in terms of achieving waste reduction targets in the future. 

The second most important aspect of a sustainable diet was judged to be “consuming Km0 or local products”. In different cultural contexts, including Spain, local products are being increasingly valued, and are associated with trust, good quality, and health [18,26]. The TNS Sofres survey (2014) [41] shows that in Spain, the three elements that matter the most in consumer evaluation of food quality are, in order of importance, the origin and place of production, product appearance, and place of purchase. The growing interest in local products is a reaction to the transformations wrought by food modernity, marked by an industrial food system that weakens the links between food and territory and between consumers and food [40,60]. “Eating local” is an attempt to return to the traditional ways and know-how that individuals are afraid of losing in a globalized society [65], and a means of rediscovering a sense of security regarding modern food [40]. Interestingly, in their perceptions of what is needed to eat sustainably, the students placed a great deal of importance on where the product comes from, but this aspect was weakly associated with their food choices, confirming the discrepancy between perceptions/norms and practices.

Finally, the third most important aspect associated with a sustainable diet was “consuming fresh and seasonal products”. As pointed out by other studies, these two characteristics may also be associated with the valuation of proximity and natural and artisanal products, as opposed to what is perceived as industrialized, transformed, chemical, toxic, and coming from a distant and unknown territory [18,26]. Food freshness is valued by the Spanish population as an attribute of food quality and a healthy diet [26,28,43,66]. A Eurobarometer survey of the public perception of food risks found that freshness is the most important food concern among Spanish consumers [28]. According to Garcia-Gonzalez et al. (2020) [20], an “abundance of fresh products” is the factor the Spanish most relate with a sustainable diet. 

### 4.2. Social Perceptions According to Gender

Gender analysis revealed that the topic of food sustainability may be more internalized by women than by men. Female participants were statistically more likely to describe their diet as vegetarian/vegan and they had a greater tendency to regard their dietary practice as responsible and socially committed or ecological and natural. The analysis of the aspects influencing food choices also showed that “ecology, environment, and animal welfare” was more important for women than men. In the analysis of food-related concerns, women expressed higher levels of concern for most of the proposed elements, including those directly associated with sustainability.

In almost all the questions in the questionnaire and in the focus group discussions, women were more appreciative of vegetarian diets and concerned about animal welfare, in agreement with other studies that report a higher prevalence of vegetarianism in the female collective [67]. In Spain, more than two out of three vegetarians are female [68]. One of the multiple motivations for vegetarianism is the impact food has on the environment [68]. Russel et al. (2021) [23] described vegetarianism as having a positive influence on sustainability attitudes and behaviors, including a preference for foods grown with sustainable agricultural practices. In a study of dietitians, those who were vegetarian were more likely to be involved in activities that promote climate change mitigation [16].

This differential attitude between female and male participants may be related to historically constructed gender roles. From childhood, through the socialization process, men, and women incorporate different norms, values, and roles that shape their female and male identities, as well as their actions in society [69]. Empathy and caring, especially through food, are values that are more associated with femininity. Women also have a greater tendency to seek information about food and nutrition and to undertake more diets [47,48]. Furthermore, there is a “gendered” division of food arising from socially constructed gender roles: meat, especially red meat, is associated with masculinity, whereas vegetables are associated with femininity [40,70]. Therefore, women are more likely to be concerned about aspects related to the environment, animal welfare, and health, as found in the present study. Garcia-Gonzalez et al. (2020) [20] also verified that Spanish women attach more importance than men to the sustainability of the food they buy. Finally, it is noteworthy that although men and women differed in their perceptions of sustainability issues, a more homogeneous discourse emerged regarding the actions required for a sustainable diet, revealing a generalized perception and strong consensus on this topic.

Despite the relevance of this work, especially in Spain, some methodological limitations should be emphasized. First, this study was carried out with students from a single academic institution in the Barcelona region, so it would be of interest to expand the sample to include other institutions and geographical contexts. Likewise, the convenience sampling of the research may entail a risk of bias because the participants may share a certain profile or interests related to the topic. Also, the qualitative phase of the study was based only on two focus groups, and more in-depth data could have been obtained with semi-structured interviews.

The authors should discuss the results and how they can be interpreted from the perspective of previous studies and of the working hypotheses. The findings and their implications should be discussed in the broadest context possible. Future research directions may also be highlighted.

## 5. Conclusions

This is the first study to analyze perceptions related to food sustainability among college students in the field of food science in Spain. Understanding social perceptions among this group is crucial to produce knowledge that can be applied to improve their academic training, to foster a critical perspective of the food system, and to promote sustainability. Although the students expressed concern about sustainability-related issues, at least in their conceptions, their eating practices were mainly associated with or influenced by taste/pleasure, health, and nutrition. Gender differences were identified, showing that the topic of food sustainability may be more internalized by women than men. Regarding the question of what constitutes a sustainable diet, the generalized view of the student population, regardless of the degree studied or gender, was that sustainability is primarily associated with environmental aspects (not wasting food, consuming Km0 or local products, and consuming fresh and seasonal products), with social and economic dimensions occupying a minor role. 

Awareness of food sustainability issues was not significantly higher among the more advanced students compared to those in the 1st year, indicating their perceptions had not been changed by training. Therefore, as academic background influences professional practices, there is a need to promote the concept of sustainability in all its complexity and multidimensionality among food science students. This lack of holistic conceptualization also calls for the development of actions that bring sustainability closer to the social practices of students. Academic training is a privileged space for the implementation of strategies that pursue these objectives. Given that many of the students are likely to find employment in different sectors of the food system, food sustainability should be discussed during their training in a more holistic, transdisciplinary, and intersectoral way. To adequately address food sustainability in university curricula, the teaching staff also require continuous training [22,23]. Additionally, building more synergies with social science disciplines would allow students to incorporate a more complex view of diet and sustainability. Finally, initiatives that bring academic practices closer to sectors outside the university, especially non-profit organizations or public policies, would be of great interest. 

## Figures and Tables

**Table 1:** General characteristics of the college students enrolled in bachelor’s degrees in Human Nutrition and Dietetics (HND) and Food Science and Technology (FST).

	Total (*n* = 300) *n* (%)	HND (*n* = 151) *n* (%)	FST (*n* = 149) *n* (%)
**Year of training**			
1st	96 (32.0%)	47 (31.1%)	49 (32.9%)
2nd/3rd	120 (40.0%)	61 (40.4%)	59 (39.6%)
4th	84 (28.0%)	43 (28.5%)	41 (27.5%)
**Gender**			
Female	241 (80.3%)	132 (87.4%)	109 (73.2%)
Male	58 (19.3%)	18 (11.9%)	40 (26.8%)
Other	1 (0.3%)	1 (0.7%)	0
**Household income**			
<999 €/month	22 (7.3%)	15 (9.9%)	7 (4.7%)
1.000–1.999 €/month	100 (33.3%)	38 (25.2%)	62 (41.6%)
2.000–4.999 €/month	158 (52.7%)	85 (53.3%)	73 (49.0%)
>5.000 €/month	20 (6.7%)	13 (8.6%)	7 (4.7%)
**Parental level of education**			
Elementary school	9 (3.0%)	7 (4.6%)	2 (1.3%)
Secondary school	31 (10.3%)	14 (9.3%)	17 (11.4%)
Sixth form	88 (29.3%)	46 (30.5%)	42 (28.2%)
Bachelor’s degree	140 (46.7%)	70 (46.4%)	70 (47.0%)
Master’s degree	32 (10.7%)	14 (9.3%)	18 (12.1%)
**Employment situation**			
Employee	92 (30.7%)	53 (35.1%)	39 (26.2%)
Internships	30 (10.0%)	20 (13.2%)	10 (6.7%)
Unemployed	178 (59.3%)	78 (51.7%)	100 (67.1%)

**Table 2:** Frequency (%) of word categories associated with “food” by the college students enrolled in bachelor’s degrees in Human Nutrition and Dietetics (HND) and Food Science and Technology (FST) through the free-association task. * Indicates statistically significant differences in responses between collectives.

	Total	Bachelor’s Degree				Gender			
		NHD (%)	FST (%)	χ2	*p* Value	Female (%)	Male (%)	χ2	*p* Value
Food (e.g., eat, food product)	28.1	19.3	36.9	11.66	0.001 *	28.7	25.9	0.177	0.674
Hedonic and gustatory dimension (e.g., pleasure, tasty, enjoy, happiness)	35.1	41.3	28.9	4.91	0.026 *	34.6	37.9	0.250	0.617
Health	5.7	8.0	3.4	2.95	0.085	6.3	3.4	0.672	0.412
Nutrition/nutrients (e.g., nutrition, energy, nutritious)	13.7	12.0	15.4	0.78	0.375	12.9	17.2	0.757	0.384
Vital aspect/need (e.g., hunger, need, survival)	12.7	12.7	12.8	0	0.964	12.1	13.8	0.134	0.715
Other (e.g., stress, habit)	4.7	6.7	2.7	2.61	0.105	5.4	1.7	1.41	0.235

**Table 3:** Aspects influencing food choices of college students pursuing bachelor’s degrees in Human Nutrition and Dietetics (HND) and Food Science and Technology (FST), showing the distribution of responses. * Indicates statistically significant differences in responses between collectives.

Aspects Influencing Food Choices	Total (%)	Bachelor’s Degree				Gender			
		HND (%)	FST (%)	χ2	*p* Value	Female (%)	Male (%)	χ2	*p* Value
Nutritional composition of foods	24.5	26.8	22.1	2.62	0.105	23.2	30.2	3.60	0.058
Eating/preparation facility	8.0	7.3	8.7	0.463	0.496	7.5	10.3	1.15	0.284
Time of availability	5.5	6.0	5.0	0.263	0.608	6.2	2.6	2.52	0.112
Price	6.7	7.3	6.0	0.402	0.526	6.0	9.5	1.94	0.164
Concern for body image	5.2	3.6	6.7	3.05	0.081	4.4	7.8	2.40	0.122
Ecology, environment, and animal welfare	8.4	7.3	9.4	0.963	0.327	9.8	2.6	6.89	0.009 *
Place of provenance/origin	3.4	4.0	2.7	0.801	0.371	3.7	1.7	1.21	0.271
Pleasure and taste preference	25.9	26.5	25.2	0.210	0.647	26.3	24.1	0.366	0.545
Disease prevention/health effects	5.2	7.0	3.4	4.19	0.041 *	5.6	3.4	0.933	0.334
State of mind	4.5	1.3	7.7	14.97	0.000 *	3.9	6.0	1.03	0.310
Composition of foodstuffs with respect to chemical additives (preservatives, etc.)	3.0	3.0	3.0	0.001	0.977	3.3	1.7	0.841	0.359

Observation: Participants could choose two response options.

**Table 4:** Perceptions of what constitutes a sustainable diet among college students pursuing bachelor’s degrees in Human Nutrition and Dietetics (HND) and Food Science and Technology (FST) according to gender and showing the distribution (%) of responses. * Indicates statistically significant differences in responses between collectives.

Aspects That Constitute a Sustainable Diet	Total (%)	Bachelor’s Degree				Gender			
		HND (%)	FST (%)	χ2	*p* Value	Female (%)	Male (%)	χ2	*p* Value
Consuming Km0 or proximity products	18.9	20.1	17.7	1.60	0.205	19.2	17.8	0.341	0.559
Consuming organic products	4.3	3.1	5.4	3.17	0.075	4.0	5.2	0.512	0.474
Not wasting food	22.5	22.5	22.4	0.006	0.936	21.7	25.3	2.44	0.119
Following a Mediterranean Diet	1.45	1.1	1.8	0.766	0.381	1.0	2.9	3.97	0.046 *
Using biodegradable or compostable materials	9.0	7.3	10.7	4.08	0.043 *	8.9	9.8	0.180	0.672
Following a vegetarian diet and/or reducing consumption of animal products	10.0	11.3	8.7	2.06	0.151	1.1	5.7	5.66	0.017 *
Consuming fair trade products	2.4	2.2	2.5	0.067	0.796	2.6	1.1	1.41	0.235
Reducing the consumption of industrial products	5.0	5.1	4.9	0.013	0.910	5.0	5.2	0.012	0.912
Being part of a consumer group/consumer cooperative	0.2	0.4	0	1.99	0.159	0.3	0	0.485	0.486
Shopping in the neighborhood market or stores	6.1	5.7	6.5	0.525	0.615	5.7	8.0	1.58	0.209
Growing/producing your own food	1.8	1.5	2.0	0.293	0.588	2.1	0.6	1.87	0.172
Buying products directly from the producer	1.7	1.8	1.6	0.057	0.812	1.7	1.7	0.004	0.952
Consuming fresh and seasonal products	16.9	17.9	15.9	1.08	0.299	16.9	16.7	0.007	0.932

Observation: Participants could choose three response options.

## Data Availability

Not applicable.

## Supplementary Materials

The following supporting information can be downloaded at: https://www.mdpi.com/article/10.3390/foods12050917/s1, Questionnaire designed for the study on the food perceptions among college students of Human Nutrition and Dietetics and Food Science and Technology.
