# Supplementary material for: Perceptions of Food among College Students in the Field of Food Science: A Food Sustainability Approach
Source: Foods. 2023 Feb 21;12(5):917. doi: 10.3390/foods12050917 (PMC10000850; doi:10.3390/foods12050917)
Supplement: Supplementary file 1 [file foods-12-00917-s001.zip › foods-2142205-supplementary.pdf]

STUDY ON THE FOOD PERCEPTIONS AMONG COLLEGE STUDENTS OF HUMAN NUTRITION AND DIETETICS AND FOOD SCIENCE AND TECHNOLOGY IN BARCELONA (SPAIN)

1. Informed consent

This questionnaire is part of an interdisciplinary study carried out by anthropologists, nutritionists and psychologists of the Food Observatory and the Food and Nutrition Campus of the University of Barcelona, which aims to compare and understand the food perceptions of Human Nutrition and Dietetics (HND) and Food Science and Technology (FST) college students at the University of Barcelona.

The questionnaire takes a maximum of 15 minutes to complete, and participation is anonymous, voluntary and without compensation. Those taking part are free to stop participating at any time. The decision not to participate or to cease participating does not imply any prejudice or coercion. In addition, taking part in the study poses no risk to the physical, social, and psychological integrity of participants. More information about the study can be requested at any time by sending an email to: info@odela.org By giving informed consent, the participant certifies that they have received sufficient information about the study, that they have read and understood the above information, and that they agree to participate in the study voluntarily.

2. Degree

- HND
- FST

3. Current year of degree

- 1st year
- 2nd year
- 3rd year
- 4th year

4. Age (years)

5. Gender identified with

- Female
- Male
- Other (specify)

6. Nationality

7. Marital status

- Single
- Married
- Divorced
- Other (specify)

8. Municipality of residence

9. Number of household members (counting the interviewee)

10. Type of cohabitation (if there is more than 1 household member)

- Relatives
- Couple
- Shared flat
- Other (specify)

11. What is the educational level of your parents? (Your answer may include up to two options)

- Primary
- Secondary
- Baccalaureate or equivalent
- Incomplete higher education
- Complete higher education
- Postgraduate (Master's/PhD)

12. In what range does the monthly income of your household fall?

- Up to €499
- €500 to €999
- €1,000 to €1,499
- €1,500 to €1,999
- €2,000 to €2,499
- €2,500 to €2,999
- €3,000 to €4,999
- More than €5,000

13. Do you work?

- Yes, full time
- Yes, part-time
- Paid internship
- Unpaid internship
- No

14. What is your current weight? (Kg)

15. What is your height? (Meters, e.g., 1.58)

16. What word do you associate with the concept of "food"? (One word only)

17. For you, food is first and foremost… (choose two options from the following list)

- A source of nutrients and energy
- A way to stay healthy
- A basic need of an organism
- A way to prevent disease
- A source of emotional well-being
- A source of pleasure
- A way of sharing
- A way to control my weight and body shape
- A political act
- An essential part of my culture

18. To what extent do you agree with the following definitions of "food"? (Strongly disagree, somewhat disagree, neither agree nor disagree, somewhat agree or strongly agree):

- Food is any substance, natural or processed, that provides nutrients to ensure the maintenance and growth of tissues and the continuity of vital functions.
- Food is life, energy, and the driving force of our body, generating physical, mental and emotional health.
- When you consume food, you are feeding on symbols, myths, and values.
- To be considered as food, a natural product must have meaning in a complex system of communication, situations, images, and behaviors.

19. What type of diet do you identify with? (Strongly disagree, somewhat disagree, neither agree nor disagree, somewhat agree or strongly agree)

- Light
- Healthy
- Pleasant and convivial
- Mediterranean
- Vegetarian / Vegan
- Ecological and natural
- Gourmet
- Traditional
- Responsible and socially committed
- Weight loss / Fitness
- Flexitarian

20. Which two of the following factors most influence your food choices?

- Nutritional composition of food
- Ease of eating/preparation
- Available time
- Price
- Concern for body image
- Ecology, the environment, and animal welfare
- Place of provenance/origin
- Pleasure and taste preferences
- Prevention of illness/ health effects
- State of mind
- Content of chemical additives (preservatives, etc.)

21. For you, the pleasure of food is related to (Strongly disagree, somewhat disagree, neither agree nor disagree, somewhat agree or strongly agree):

- Savoring the taste of food
- Cooking
- Sharing meals
- Buying food
- Growing food
- Satisfying hunger
- Discovering new gastronomic cultures / new flavors
- Eating healthily
- Controlling food intake

22. Which two of the following do you most associate with "cooking"?

- Sharing
- Obligation
- Pleasure
- Art
- Health
- Sustainability
- Effort
- Care
- Waste utilization

23. Chose three of the following options as ways to promote and guarantee "food sustainability"

- Consuming Km0 or local products
- Consuming organic products
- Not wasting food
- Following a Mediterranean diet
- Using biodegradable or compostable materials
- Following a vegetarian diet and/or reducing consumption of animal products
- Consuming fair trade products
- Reducing consumption of industrial products
- Joining a consumer group/cooperative
- Shopping in a market or neighborhood shops
- Growing/producing your own food
- Buying food directly from the producer
- Consuming fresh and seasonal products

24. To what extent do you agree with the following sentences? (Strongly disagree, somewhat disagree, neither agree nor disagree, somewhat agree or strongly agree)

- I am in good health
- My diet is healthy
- My diet can affect my health
- It is easy to have a healthy diet
- Having a healthy diet is a matter of personal will
- I control my diet
- I should control my diet more
- I should do more sport
- I should lose weight
- I should gain weight
- I have a troubled relationship with my body
- I often feel guilty about eating
- My body shape and weight are important factors in my future profesional activity

25. Choose the three options you think are the most important for a healthy diet

- Eating fresh/natural foods
- Eating with pleasure
- Paying attention to the physiological signals of hunger and satiety
- Having contact with food and cooking
- Eating according to the Food Pyramid
- Having a varied diet, a bit of everything
- Preferably consuming organic food

- Eating at the table without looking at the television, computer or smartphone
- Eating in the company of other people
- Consuming the full range of macronutrients, vitamins and minerals
- Having a balanced diet
- Eating at the main mealtimes without snacking between meals
- Consuming 5 servings of fruits and vegetables a day
- Eating a vegetarian diet or a diet low in animal products

26. In your opinion, how important are the following for a healthy diet? (Not important, not very important, quite important, important or very important)

- Eating fresh/natural foods
- Eating with pleasure
- Paying attention to the physiological signals of hunger and fullness
- Having contact with food and cooking
- Eating according to the Food Pyramid
- Having a varied diet, a bit of everything
- Preferably consuming organic food
- Eating at the table without looking at the television, computer or smartphone
- Eating in the company of other people
- Consuming the full range of macronutrients, vitamins and minerals
- Having a balanced diet
- Eating at the main mealtimes without snacking between meals
- Consuming 5 servings of fruits and vegetables a day
- Eating a vegetarian diet or a diet low in animal products

27. To what extent do the following prevent you from following a healthy diet? (Not at all, a little, moderately, quite a lot or very much)

- The pleasure of eating and my food preferences
- Lack of information on healthy eating
- Lack of culinary knowledge
- The traditional food habits of my culture
- The eating habits of my family
- Difficulty of combining a healthy diet with my social life
- Lack of time to buy, cook and eat food
- The price of food products regarded as healthy
- Lack of facilities/space at home to store and prepare food
- Lack of self-control and discipline
- Lack of will-power
- My emotional states
- Lack of autonomy

28. Rate each of the following foods on a scale of 1 to 5 according to how tasty you think they are (1: not very tasty, 5: very tasty)

- Red meat
- White meat
- Vegetables
- Fish
- Seafood
- Meat derivatives
- Dairy products and derivatives
- Fruits

- Cereals
- Legumes
- Nuts
- Olive oil
- Eggs
- Sweets
- Ultra-processed foods (snacks, pastries, etc.)
- Sugary drinks (soft drinks)
- Wine
- Beer
- Fermented foods (excluding alcoholic beverages)

29. Rate the following foods on a scale of 1 to 5 according to how healthy you think they are (1: not healthy, 5: very healthy)

- Red meat
- White meat
- Vegetables
- Fish
- Seafood
- Meat derivatives
- Dairy products and derivatives
- Fruits
- Cereals
- Legumes
- Nuts
- Olive oil
- Eggs
- Sweets
- Ultra-processed foods (snacks, pastries, etc.)
- Sugary drinks (soft drinks)
- Wine
- Beer
- Fermented foods (excluding alcoholic beverages)

30. How important do you think the following attributes are for a food to be regarded as healthy? (Not important, not very important, quite important, important or very important)

- Low in fat
- Without sugar
- Gluten-free
- Lactose-free
- Low in calories
- Wholegrain
- Vegetable and of non-animal origin
- Respects animal welfare
- Natural
- Fresh
- Industrial
- Locally produced/Km0
- Rich in vitamins, minerals and other essential nutrients
- Seasonal
- Ecological

- Cooked at home
- Free of preservatives or sweeteners
- Complies with sanitary controls
- Prevents chronic diseases
- Non-fattening
- Pleasant/tasty
- Traditional
- Of known origin
- Is produced by yourself
- Not genetically modified

31. To what extent do the following give you confidence about food quality? (Not at all, a little, moderately, a lot or very much)

- Brand
- Origin/place of production
- Product appearance
- Type of packaging
- Place of purchase
- Type of ingredients
- Food quality designations
- Price
- Form of preparation/production
- Date of expiry

32. How worried are you about the following? (Not at all worried, not very worried, quite worried, worried or very worried)

- Pesticides in fruits, vegetables and cereals
- Unhygienic conditions at home
- New viruses (eg., bird flu, COVID-19)
- Allergic reaction
- Residues in meat such as antibiotics and hormones
- Contamination in fish (mercury, microplastics)
- Fattening
- Hygienic conditions outside home (restaurants, shops, etc.)
- Chemicals formed during heating, baking, grilling, or frying
- Bacterial contamination such as Salmonella in eggs or Listeria in cheese
- Concentration of pollutants such as mercury and dioxins
- Mad cow disease (BSE)
- Animal welfare
- Animal cloning
- Genetically modified (transgenic) products
- Chronic non-communicable diseases (diabetes, cardiovascular diseases, cancer, etc.)
- Obesity
- Additives such as colorants, preservatives or flavorings used in food or beverages.
- Food waste
- Use of plastic and plastic packaging
